# Restricting family life - an examination of citizens’ views on state interventions and parental freedom in eight European countries

**DOI:** 10.1080/13691457.2023.2227772

**Published:** 2023-07-10

**Authors:** Marit Skivenes, Asgeir Falch-Eriksen, Bilal Hassan

**Affiliations:** a Department of Government, Centre for Research on Discretion and Paternalism, University of Bergen, Bergen, Norway; b Department of Social Sciences, OsloMet, Oslo, Norway

**Keywords:** Child protection, comparative, family, freedom, parental rights, barnevern, komparativ, familie, frihet, foreldres rettigheter

## Abstract

This paper examines the public views – a total of 10,348 persons – on restrictions of personal autonomy of others to protect the interest of children. We use representative country samples of the adult populations of Austria, England, Estonia, Finland, Germany, Ireland, Norway, and Spain, and ask them to consider an experimental vignette with three different parental conditions: substance abuse, mental health problems, and learning difficulties. The findings display that most people would restrict parental freedom to protect the child, and a stricter restriction when the parent struggles with substance abuse compared to mental health compared to learning difficulties. There are some country differences, and when examining the role of institutional context of child protective system, a correlation is detected with significant differences between population views in a right-oriented system versus a well-being system and maltreatment system. In light of the ongoing European debates about child protection and how controversial and contested this area of the welfare state seem to be, it is interesting to learn (also) from this study that people, across countries, individual differences, child protection systems, overall are supportive of state intervention and support in a situation with a child at potential risk.

## SAMMENDRAG

Denne artikkelen undersøker befolkningens synspunkter – totalt 10 348 personer – på begrensninger av individuell autonomi for å beskytte barns interesser. Vi har representative utvalg av voksenbefolkningen i Østerrike, England, Estland, Finland, Tyskland, Irland, Norge og Spania. De er bedt om å vurdere en eksperimentell vignett med tre ulike foreldreutfordringer som har betydning for utøvelse av foreldreskap: rusmisbruk, psykiske helseproblemer og lærevansker. Resultatene viser at de fleste respondenter ønsker å begrense foreldrenes frihet for å beskytte barnet, og at begrensningene er strengere når foreldrene sliter med rusmisbruk sammenlignet med psykiske helseproblemer og lærevansker. Det er noen landsspesifikke forskjeller. Institusjonelle forhold kaster lys over landforskjeller, ved at det er betydelige forskjeller mellom befolkningens syn i et barnevernsystem som er barnerettighetsorientert, kontra et barnevernsystem som er orientert mot å beskytte mot mishandling. I lys av de pågående europeiske debattene om beskyttelse av barns rettigheter og familier, og hvor kontroversiell og omstridt barnevern synes å være, er det interessant å lære (også) fra denne studien at folk på tvers av land, individuelle forskjeller og barnevernsystemer, generelt støtter barnevernets inngrep og bistand i en situasjon der et barn er i potensiell risiko.

## Introduction

In this paper, we examine citizens’ views on child protection, an area of state responsibility that seems to be contested in many countries, and in which governments must conduct a balancing act between the rights of parents and the rights of children. Basic to constitutional democracies are individual rights to private autonomy and protection from harm from majority rule or unlawful treatment. Rights thereby constitute an area for constitutional review and for governmental vigilance throughout the democratic chain of command from parliament to street-level and frontline bureaucrats. For our purposes, rights are legal principles that establish various arenas in which citizens and individuals can make free choices in their ‘pursuit of happiness’ without interference. However, the flipside to these freedoms, and acutely relevant for child protection, is that they are only legally valid as long as they do not infringe upon the freedom of others or do them harm (Mill, 1903). Through legislation, jurisprudence, and constitutional review, individual freedoms are maintained and tested. Whereas private autonomy is fundamental to constitutional democracies, rights are further controlled, enforced, maintained, and developed by the republican ethos of self-government. Human rights are also valid for children, as the UN Convention on the Rights of the Child (CRC) (1989) clearly shows. This convention has been ratified by all countries in the world, except the United States, and Article 19 requires governments to intervene when children experience maltreatment or neglect:

States Parties shall take all appropriate legislative, administrative, social and educational measures to protect the child from all forms of physical or mental violence, injury or abuse, neglect or negligent treatment, maltreatment or exploitation, including sexual abuse, while in the care of parent(s), legal guardian(s) or any other person who has the care of the child. (CRC, Article 19 (1))

We study what type of intervention people in eight European societies with contrasting child protection systems recommend when a child is potentially at risk, and if degree of intervention varies with the type of parental problem. We use representative country samples of the adult populations of Austria, England, Estonia, Finland, Germany, Ireland, Norway, and Spain.

There are multiple ways to legitimise intervention with parents, including religious, philosophical, moral, and epistemic doctrines that may guide state willingness to restrict the personal autonomy of others to protect the interest of children (see, for example, Berrick et al., 2023). Individuals’ views on child protection and governmental responsibility may be driven by various factors, such as core values concerning family and security, confidence in the state, rights orientation, institutional context, or individual responsibility. The focus of our analysis in this paper is fourfold. First, what are people’s views on government responsibility for children in a situation of potential risk? Second, do people differentiate concerning what the government should do based on the type of parental behaviour that entails risk to the child? Third, are people’s views and attitudes correlated with the institutional context of their state and government in which they are embedded? Fourth and finally, are there variations between people that are correlated with their sociodemographic backgrounds?

This study contributes to existing research on the legitimacy of state interventions into citizens’ family lives, and to the knowledge base on how populations interpret the CRC and Article 19. We present results concerning discussions of institutional context and people’s relative weighting of personal responsibility against traits that are beyond their control. The paper narrows knowledge gaps about how principles of nonintervention in private matters are considered in child protection situations. Finally, the paper provides insight into the views of citizens in various countries on public responsibility for children and the threshold for interventions.

The structure of the paper is as follows. In the next section, we present the knowledge platform and hypotheses, followed by sections on methods, results, and discussion. In an Online Appendix Supplementary Material is provided to ensure transparency and accountability in research.

## Knowledge platform and hypotheses

Government responsibilities for protecting children’s rights and securing children from maltreatment and abuse are supported and enforced in most high-income countries. However, how much protection that is considered necessary is interpreted in various ways across countries, as reflected in different thresholds for placing children in out-of-home care (Berrick et al., 2023; Gilbert et al., 2011), in studies of differences between frontline workers and judicial decision-makers’ assessments of similar cases (Berrick et al., 2019), and in studies of social workers’ opinions (Berrick et al., 2015, 2016; Kriz & Skivenes 2013, 2014, 2015). There are also indications that views differ within the same country and between countries on which type of circumstances that requires government intervention, which rights to protect, and how much the state should and can do (Berrick, 2011; Berrick et al., 2022; Skivenes, 2023; Skivenes & Benbenishty, 2023; Skivenes & Thoburn, 2017; Benbenishty & Schmid, 2013; Burns et al., 2021; Schmid & Benbenishty, 2011). Based on the main findings from this literature, the first two hypotheses are simply:

H1: Populations will support government intervention and restrictions of parental freedom in a situation of potential child maltreatment, abuse, or neglect.

H2: There will be differences between countries and between individuals in their degree of support for government intervention and restriction of parental freedom.

This study explores people’s willingness to restrict or revoke personal autonomy whenever another person’s health and safety are at stake, which in our case is a child’s risk of detriment from parental care. Whenever children need protection, it is from some type of detriment caused by the caretaker, such as neglect, violence, or various types of abuse. We examine potential child neglect, as it is open to discretionary decision-making whereby acceptable care may be within reach. An experimental vignette survey isolates the impact of types of parental challenges that may prompt respondents to consider restricting personal autonomy through degrees of revoking the right to parent. Substance abuse, mental health problems, and learning difficulties are all common reasons for coercive interventions by the child protection system, and specifically studies of judgements about care-order removals in the eight European countries discussed in this paper show that these three parental challenges characterise interventions in cases about newborn children (Skivenes, 2023; Luhamaa et al., 2021). We test whether the type of parental problems matter for people’s willingness to restrict autonomy based on cultural beliefs about what is within and beyond a person’s control. We approach this by assuming that an individual may be perceived as responsible for a social problem and able to act upon it (lifestyle health problems) or it can be perceived to be outside a person’s control (e.g. a learning disability). Empirically, there is an explorative study of populations in four countries (England, Norway, Poland, and Romania) that reveals differences in responses concerning the appropriate degree of intervention according to the type of parental problem (unsatisfactory care, alcohol misuse, mental illness, or intellectual disability) when the child’s welfare is at stake (Skivenes, 2023).

Thus, the *third hypothesis* is that a parent’s type of problem, whether social (substance abuse), health related (mental health), or a disability (learning disability), has an impact on willingness to restrict personal autonomy when the individual has the responsibility to care for a newborn. We present the exact same situation for the child but change the parental problem in the case. In the first scenario, the mother abuses alcohol, which is depicted as a social problem. In the second scenario, the mother is suffering from a mental health problem that may be temporary in character. In the third scenario, the mother is depicted as having a lifelong learning disability. We expect most restrictions to be on the alcohol misuse scenario, followed by mental health problems, and finally intellectual disability.

H3: Respondents that review X1, substance abuse problems, more strongly favor restricting a person’s freedom than those that review X2 (mental health), and, than those reviewing X3 (learning disability).

### Institutional context

We expect there to be differences between countries, and we examine whether these differences may be explained by the child protection systems in place. The theoretical basis for this approach is policy theory, in which a child protection system is regarded as an expression of an institutional context. A premise in policy theory is that policies and welfare institutions influence citizens’ attitudes and their views on the role and status of the welfare systems (see, for example, Berrick et al., 2022; Skivenes, 2023; Svallfors, 1996, 2012; Valarino et al., 2018).^1^ The premise is that the institutional and cultural context in which people are embedded forms their views on what should be a collective responsibility and how society should be built. In welfare state literature, there is an ongoing discussion about how institutional/cultural context and individual preferences and attitudes are formed and how they relate to policy choices (Svallfors, 2012; Valarino et al., 2018). One branch of the theory argues that citizens elect their representatives, and that they determine the content of the policy institutions should implement in a country. However, another branch argues that the established institutions will also influence the popular will, and as such institutional context shed light on the opinions and attitudes of the people. For example, that the quality of institutions drives social trust (Rothstein & Stolle, 2007), and political trust as a driving force behind support for environmental policies (Harring, 2018). Family-specific societal and institutional context highlights the embedded values and understandings of children, families, and ways of acting that may align with or directly oppose the rights prescribed in documents such as the CRC. The nation-states in this study all adhere to the principle of the state monopoly on legitimate coercion, and they have all delegated authority to their respective child protection systems to reach decisions on intrusive interventions and coercion. In our study, we use the child protection system as a measure of institutional context and as an independent variable to explain differences between countries.

Child protection systems in high-income countries are usually categorised into three types based on their risk orientation, i.e. what risk circumstances that the state by its child protection system set out to protect (Berrick et al., 2023; see Gilbert et al., 2011). The three types of systems are ‘maltreatment protective systems’, ‘child well-being protective systems’, and ‘child’s rights protective systems’. A maltreatment protective system has a relatively high threshold for intervention into the family, and a primary focus to protect children’s health and safety. A child well-being protective system in addition to protect children´s health and safety, also aims to provide help and support to families to prevent and reverse negative developments. The child’s rights protective system aims to protect children’s health and safety, provide help and support, and in addition set out to protect the full range of rights that children are accorded in the CRC. It has a strong focus on children’s rights and needs, regarding children as moral individuals on par with other individuals in society. Finland and Norway are usually categorised as having a child’s rights protective system (Skivenes, 2011; Hestbæk et al., 2023; Pösö, 2011; Höjer & Pösö, 2023). Austria, Germany, and Spain are categorised as child well-being protective systems (Berrick et al., 2023). California, England, Estonia, and Ireland are typically categorised as maltreatment protective systems (Skivenes, 2011; Berrick et al., 2023; Parton & Berridge, 2011; Thoburn, 2023), although England has also been described as a hybrid system, leaning toward a child well-being system but reactive and maltreatment-oriented in high-profile cases (Thoburn, 2023).

H4: Country differences will exist along these system types;

H4-1. Populations with child’s rights protective systems (Finland and Norway) will favor restricting a parent’s freedom compared with populations with child well-being protective systems (Austria, Germany, and Spain) or child maltreatment protective systems (England, Estonia, and Ireland);

H4-2. Countries with child maltreatment protective systems will be least supportive of restricting parental rights.

### Sociodemographic variables

In welfare state research, the analysis of sociodemographic background variables has provided a wide range of correlations, and although class-related variables such as education and income are more often correlated with specific views on the welfare state, this is not a strong basis on which to build. The reason for this, as Svallfors (2012) has pointed out, is primarily the huge variation in analytical and conceptual approaches to the study of welfare attitudes. Existing research on people’s attitudes on state interventions and child protection issues concurs with welfare state research. Diepeveen et al. found that female or older respondents had greater acceptance of interventions (Diepeveen et al., 2013). Restrictive policies already in place had greater support, and policies that targeted children and young people received greater acceptance from the population (Diepeveen et al., 2013). Studies specifically on child protection systems show that gender, age, political orientation, income, and education matter: women and individuals over 55 years of age show less confidence in the child protection system (Juhasz & Skivenes, 2016), whereas people that are politically on the left, and in the high-income and high-education categories, have more confidence in the child protection system (Juhasz & Skivenes, 2016). People over 55 also have a less positive attitude toward adoptions from care (Skivenes & Thoburn, 2017; Helland et al., 2020). We expect there to be heterogeneity, and we use the background characteristics collected to identify differences between respondents. Specifically, we test whether there are differences between individuals based on their political orientation, religious identification, income, gender, age, whether they have children, and whether they are from a migrant background.

## Method

The design for this study is an experimental survey vignette to secure cross-country comparability between populations (*n* = 10,348) in eight countries – Austria, England, Estonia, Finland, Germany, Ireland, Norway, and Spain. We manipulate one variable, individual problem type, and examine whether this is causally connected to willingness to restrict parental freedom.^2^ The vignette was developed by the first author, with feedback and contributions from researchers in the eight countries as well as by a group of interdisciplinary researchers outside the research project. The vignette has also been tested on lay people. The vignette and survey questions were developed in English, then translated into the non-English languages. Translations were back translated and reviewed by researchers in the field of child protection who were native speakers. An appendix is available online to secure transparency in research and detailed overview and analysis of the data material: https://discretion.uib.no/wp-content/uploads/2023/06/Appendix.pdf.

The 10,348 respondents were recruited using the data collection agency Respons Analyse, and data were collected in January and February 2019. The respondent numbers were as follows: Austria 1033, England 1735, Estonia 1012, Finland 1008, Germany 2047, Ireland 1027, Norway 1487, and Spain 1000. The samples of respondents are nationally representative (18+ years old) on observable characteristics (gender, age, and geography, except for Estonia, where the representativeness was only controlled for in terms of gender and age). The sample was weighted to ensure representativeness. For the background questions, we used standard formulations provided by the data collection agency. We also included items on political orientation, religious belonging, and migrant background. The survey used an experimental vignette in which the parental problem was manipulated (X1 substance abuse, X2 mental health, X3 learning disability). We label them treatments. Each participant was randomly assigned one of the vignettes, X1, X2, or X3, with about 3437 participants responding to each vignette. In each country, around 333 respondents received each vignette, except for England, Germany, and Norway where around 500–700 respondents responded to each vignette. The randomisation resulted in an overall even distribution of respondents based on background characteristics (see Table A1 in the Appendix). However, there is an uneven distribution on the variables on immigrant (Austria, Estonia, and Finland), income (Spain) and religion (Ireland).

The experimental vignette reads as follows:

‘Please consider the following case. The information is based on testimonies from three professionals.

X1.

A baby is born at the hospital. The mother is 22 years old. The father is unknown. The mother has a drug and alcohol abuse problem but has refused to use the services offered to her. The mother needs prompting and help to feed and care for the baby, and the hospital staff have concerns for the baby’s safety.

The mother agreed to stay in a supervised mother–child center, but after six weeks withdraws her consent. She wants to go home with the baby and does not want any interference from professionals.

The staff at the mother–child center have serious concerns for the baby’s well-being, because the mother has not shown a noticeable change in caring for the baby and she does not provide emotional warmth and stimulation for the baby.

The mother does not have family or friends who can provide support for her and the baby.

Vignette X2 and X3 are identical, except that the underscored wording was replaced with in X2 ‘has a long-term mental health problem,’ and in X3 ‘has a mild learning disability.’ Respondents were asked to consider the information in the vignette and give their opinion on how the public authorities should proceed according to five response options:

1. The mother should be allowed to return home with the baby without any interference from professionals.
2. The mother should be allowed to return home with the baby if she accepts necessary support services.
3. The mother should stay at the mother–child centre with the baby until there is an improvement in her ability to care for the baby.
4. The mother should return home, but the baby should be placed with a foster family.
5. The mother should return home, but the baby should be placed permanently with an adoptive family.

The response options are from no interference at all (1) to a permanent separation of mother and child (5). Two options (2 and 3) restrict the mother’s expressed will about going home without interference but keep the mother and the baby together. One option temporarily separates the mother and the child (4) by placing the baby in a foster home.

In our analysis, we used Stata version 17 and weighted data. We provided a descriptive analysis of results, merging the response categories into three, with response alternatives of (1) ‘No restrictions’, (2) and (3) as ‘Some restrictions’, and (4) and (5) into ‘Removal of child’. To identify differences in percentages between countries, we used the Zigne significans data tool and tested at the 1% level. Furthermore, to examine the significance of treatment effects and correlation of background variables, the dependent variable is used as an interval scale and a chi-square test, *t*-test, and one-way analysis of variance (ANOVA) were conducted. We performed an ANOVA and Tukey *post hoc* test and conducted additional analyses to examine whether sociodemographic factors (gender, age, partnership, children, or education) played a role in explaining restrictions on freedom.

For the Ordinary Least Square (OLS) regression, we developed six ordinary least square models. Model 1 estimates the effects of learning disability, substance abuse and mental health conditions on support for restricting parental freedom. Support for reducing parental freedom was regressed on the three experimental conditions in Model 1 without taking contextual or demographic factors into account. Model 2 expands on Model 1 by including country as a variable to evaluate whether there are considerable and meaningful differences in support for restricting parental freedom across countries. Model 3 regresses support for restricting freedoms on three experimental circumstances, nine demographic variables, and institutional context (i.e. child protection systems). Models 4–6 separately tested Model 3 within each of the three different child protection systems. Results are displayed in Appendix Table A7.

We also explore if there are sociodemographic features that are related to favouring no government intervention on the one hand, and a strong intervention on the other hand. We use a simple test, examining differences in response distribution, see Appendix Table A6.

We report findings at the 0.1%, 1%, and 5% significance levels, aware that *p* < 0.05 is on the margin of statistical significance. General ethics approval for the research project was granted in accordance with Norwegian national guidelines and the General Data Protection Regulation, 2016/679. This study does not include any identifiable data on the study participants in any of the countries. For general information on our use of survey data providers, see: blinded version attached for reviewers.

### Limitations

This is a comprehensive and unique survey material, but survey studies also have limitations. Although careful attention has been devoted to translations, the interpretations of the meaning of the vignette and the response options may differ. The survey may have biases of which we are unaware, and as in all opinion surveys, the representativeness of samples is based on selected variables and may thus not sufficiently include all subgroups in a population. Vignettes, although realistic, are still fictitious, and respondents’ recommendations may differ from those in a real-life situation.

## Findings

The analysis confirms the two first hypotheses on a majority recommending restrictions to the mother’s freedom and that there will be variations between countries. A large majority of respondents (81.2%, see Table 1) chose one of the two options that somewhat restrict the mother’s freedom, either by allowing her to return home if she accepts support and services (34.4%), or by keeping the mother at the mother–child centre (46.8%); see Appendix Table A2. Very few citizens, 3.6%, were willing to accommodate the mother’s wish to go home without any support or interference, and equally, few (5.6%) suggested that the baby should be placed permanently with an adoptive family (see Table A2). There are a range of significant country differences between the respondents that suggested ‘No restrictions,’ ‘Some restrictions,’ and ‘Removal of child’ (see Table A3(a–c) in Appendix for details). For example, 5.9% of the German respondents but only 1.4% of the Norwegians suggest no restrictions on the mother. A large majority (87.3%) of the respondents in Austria and Estonia suggest ‘Some restrictions,’ whereas among the Norwegians and Finns, the proportions are 78% and 76.2%, respectively. In Austria, 9.2% of those surveyed suggest removal of the child, in contrast to 22.4% of those in Norway.

**Table 1.** Descriptive results.

	No Restriction (Value 1)		Some Restrictions (Values 2 and 3)		Removal of Child (Values 3 and 4)	
	*%*	N	*%*	N	*%*	N
Austria	3.49	36	87.70	910	8.81	91
Estonia	2.66	46	78.62	1370	18.72	326
Finland	2.57	25	86.93	843	10.50	102
Germany	4.18	42	78.13	791	17.69	179
Ireland	6.07	125	80.74	1660	13.19	271
Norway	4.27	44	82.99	856	12.73	131
Spain	1.34	20	76.39	1140	22.27	332
UK	4.08	41	82.95	833	12.97	130
Total	3.67	380	81.22	8404	15.11	1564

Total *N* = 10,348. Percentages and *n*. Merged responses 2 and 3, and 4 and 5. See Table A2 in Appendix for percentages and *n* on each response alternative.

### Treatment effects

**Figure 1.**
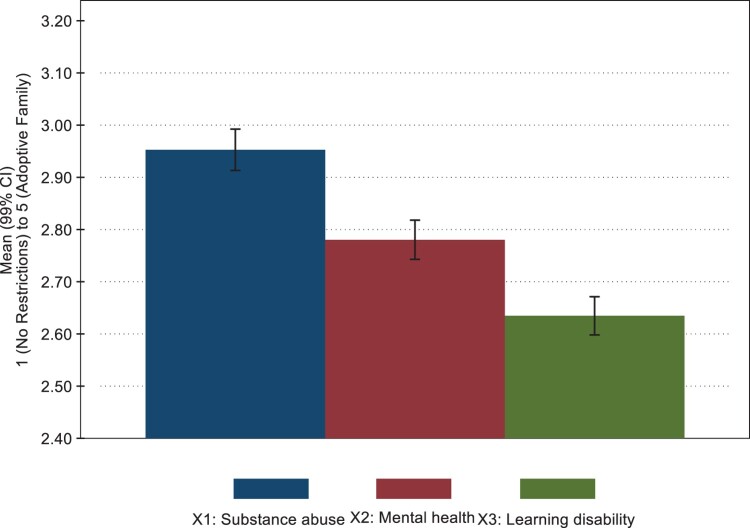
Support for restricting a mother, testing for treatment effects. Mean values. 99% CI. *N* = 10,348.

Hypothesis 3 about the impact of parental problems is also supported. We examined whether the type of parental challenge mattered for the choice of restriction, distinguishing between the following three treatments: substance abuse, mental health problems, and intellectual disability. The analysis display treatment effects (see Figure 1 below, and A4 in Appendix). Using mean values and anticipating that the five options represent increasing levels of intervention, we detected a significant difference between the three parental scenarios. Treatment effects are also tested in a Tukey *post hoc* test, showing that support for restricting parental freedom is highest among the respondents in the substance abuse treatment group (F(2,10345) = 112.70, *p* < .001), followed by the mental health group (mean difference = −0.18, *p *= 0.011) and the learning disability group (mean difference = −.26, *p *< .001) (see also Table A5 in Appendix).

**Figure 2.**
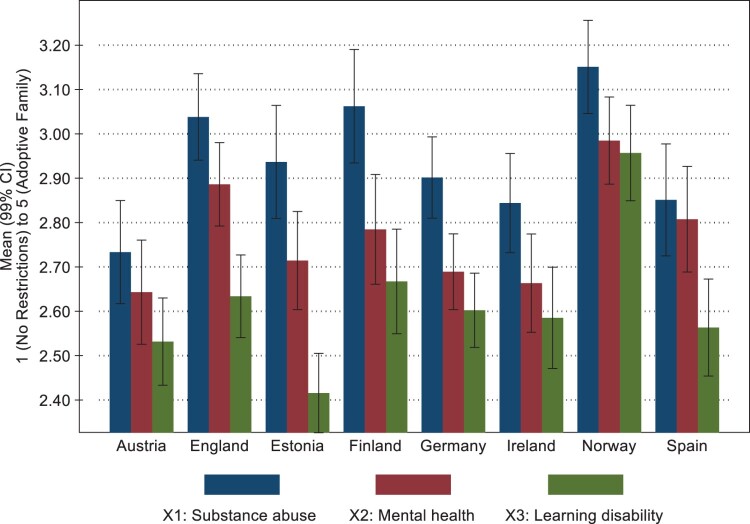
Between-Country differences in support for restrictions of mother, testing for treatment effects. Mean values. 99% CI. *N* = 10,348.

We examined whether the treatment effects for the total sample were manifested at the country level, finding they were only significant for some countries and between some treatments (see Figure 2). In all countries, people favour more restrictions on a parent with a substance abuse problem, and in Finland and Germany, people placed significantly stricter restrictions in the substance abuse scenario compared with the mental health and learning disability scenarios. For three countries – England, Estonia, and Spain – there is a treatment effect for substance abuse and mental health on the one hand and the learning disability on the other (see Figure 2 and Table A5 in the Appendix). We find the same pattern with decreasing willingness to intervene regarding X1–X3 for all countries except Norway, where there is very little difference between X2 and X3, and Spain, where there is little difference between X1 and X2 (see Figure 2 and Table A5 in the Appendix).

## Institutional context

Examining whether country differences may be explained by institutional context, which for our purpose is the type of child protection system, we identify a significant and positive correlation between the system and degree of intervention (see Table 2). Respondents in children’s rights systems (Finland and Norway) have more favourable views of interventions and restrictions of parental freedom (mean 2.96) compared with those in child well-being systems (Austria, Germany, and Spain) (mean 2.71). However, maltreatment-oriented systems are more supportive of restrictions on parental rights (England, Estonia, and Ireland) (mean 2.77), than well-being systems. The results of the ANOVA test to find out if results are significant, confirmed that there are significant differences (*F*(2,10345) = 63.68, *p *= 0.000). Results from a Tukey *post hoc* test which examines pairwise comparisons, indicate that the systems differ, although the difference between child maltreatment and well-being systems is very marginal (see Table 2).

**Table 2.** Comparison of mean scores by institutional context using Tukey *post hoc* test.

System	Mean	*F*-statistics	Mean difference		99% CI	
					Lower bound	Upper bound
Child maltreatment (*M*)	2.76	*F*(2,10345)	W vs M*	−0.05	−0.11	0.00
Well-being (*W*)	2.71	=63.68***	R vs M***	0.19	0.12	0.25
Child rights (*R*)	2.95		R vs W***	0.24	0.18	0.31

Note: *p ≤ .05; ***p ≤ .001.

## Demographic variables

We examined the relationship between sociodemographic variables and support for restricting freedom, and find an association between not having a partner, higher education, and earning a middle income (see Table A7 in the Appendix). We find a weak association between nonimmigrant respondents, political support for the government, and willingness to intervene (see Appendix, Table A7) (see also Table A7 in Appendix, Model 3). However, several of the associations lost significance when included in the models inside each of the child protection systems (see Appendix, Table A7, Models 4–6). Specifically, Models 4–6, show that under the child maltreatment system, having children (*b* = −0.11, *p* < 0.01) and being an immigrant (*b* = −0.15, *p* < 0.01) negatively affect support for limiting parental flexibility. Similarly, in the child welfare system, while supporting the opposition party has a negative impact on support for restricting parental freedom (*b* = −0.13, *p* < 0.001), being an adult (*b* = 0.11, *p* < 0.05), not having a religion (*b* = 0.09, *p* < 0.01), and belonging to a high-income group have a positive impact on support for restricting parental freedom. Finally, under the child right system, being an immigrant (*b* = −0.23, *p* < 0.05), having no partner (b = −0.19, *p* < 0.01), and being male and supporting the opposition party (b = −0.07, *p* < 0.10) all have a negative impact on support for constraining parental freedom. Those with a higher education, on the other hand, favour curtailing parental freedom (*b* = 0.18, *p* < 0.001).

Exploring the background of the respondents that suggest no state intervention in a situation such as those described in the vignette (3.7% of respondents (*n* = 380)), they are more likely to be men, have a partner, have low education, being religious, and, less likely to be affiliated with the political orientation of the government (see Table A6 in the Appendix). On the other end of the spectrum, the respondents that believe the child should be permanently placed with another family (5.6% of respondents (*n* = 575)), are more likely to have a partner and not having children.

## Discussion

This paper advances our insights into an under-researched topic in social policy concerning popular views on how governments draw the line between family sovereignty, parental rights, and children’s rights in societies. Child protection is a field where the dilemma is obvious, as parents’ rights to live their lives freely and to raise their children as they see fit may conflict with the child’s rights to a healthy and safe upbringing and children’s rights as they are set out in the CRC, as well as the state’s obligation to protect children’s rights. The triangle of parties in child protection is protection rights and the family is often portrayed as the definition of a private sphere, but it is also the arena for maltreatment and oppressive practices, as clearly pointed out by feminists and children’s rights advocates (Young, 1997; see also Shapiro, 1999). The analysis of representative samples of the population in eight European countries reveals a situation of potential child neglect, and the results show us that very few people would suggest that the government should not intervene. A large majority – 8 out of 10 – suggest that the government should require the parent either to accept services or remain in the residential unit. Additionally, about 15% of respondents recommend that the child and the parent be separated temporarily or permanently. Although this pattern is evident in all countries, there are significant country differences in terms of what people would advise the government to do. For example, the Norwegians endorse interventions, and only a handful of respondents recommend that the government stay away from the family. Moreover, one out of five suggests removing the child, temporarily or permanently. By contrast, 6% of German respondents recommend that the family be left alone, and 13% would remove the child. We shall return to possible explanations for this below.

To increase our understanding and to explain some of our findings, we first ask whether the type of parental problem is important, and second whether institutional context explains some of the differences between countries. The experimental design allowed us to test whether three distinct parental problems may explain variation in support for government intervention. We have chosen drug and alcohol abuse, mental health, and mild learning disability, because the first may be regarded as being within an individual’s control, whereas a sickness or a condition is outside a person’s control. Furthermore, these three parental problems are empirically prevalent in child protection cases and are predicted to reduce parental capacities, and as such put a child – especially a newborn baby – at risk (Skivenes, 2023; Luhamaa et al., 2021; Ruiken, 2022). The results show a distinct treatment effect. People are of the opinion that drug or alcohol abuse requires stricter restrictions on parental freedom than mental health issues or a mild learning disability. We believe this is driven by a sentiment that this problem is within an individual’s control, and thus such a parent may receive more blame than those in the other two scenarios. However, there may be other explanations, such as respondents believing that substance abuse poses a greater risk to the child than mental health issues or a mild learning disability. The latter two would cover a range of potential issues that may or may not constitute a challenge for parenting a baby. The same could be said for substance abuse problems, but we anticipate that people will have a specific idea of how substance misuse (compared with the other two problems described in the vignette) affects parental capacities to care for a baby. Empirically it is estimated that about 8% of the European population misuses alcohol (see Skivenes, 2023). Although it is not mentioned in the vignette, respondents may also suspect that the child has withdrawal symptoms due to substance use during pregnancy, and thus people may think that the baby demands more attention from a parent than a baby that has not been exposed to substances. Although, we cannot say for sure what drives these findings, we believe it makes sense to pursue further research into the idea that individual responsibility may play a role in how people assess what actions are necessary and acceptable for the government. The regression analysis expands on these findings, see below.

It is however clear that the treatment effects are less evident within countries, although the same treatment pattern is visible in all country samples except for Norway and Spain. In Norway, there is little distinction between mental health and learning disabilities, and in Spain, there is little distinction between substance abuse and mental health. A possible reason for people in Austria, Ireland, and Norway not distinguishing significantly between the three types of parental problems is that they focus on the child’s circumstances and assess the situation for the child as equally acceptable or not acceptable. As a Norwegian county board judge commented in a key informant interview: ‘for the baby, the risk is equally high in all three scenarios’ (BLINDED). For two countries, Finland and Germany, populations are significantly more willing to suggest higher levels of intervention for substance abuse than for mental health and mild learning disability. It is interesting to observe that the populations in three countries – England, Estonia, and Spain – show a significant difference between substance abuse and mental health on the one hand contrasted to learning disability on the other, indicating that a mild learning disability is considered less of a reason for the government to intervene. An exploration of the policies on and about individuals with learning disability in these three countries, as well as a literature search, did not result in any plausible explanations for this finding. However, based on the author’s knowledge of different child protection systems, it may be relevant to explore if for example the English child protection system has a different approach to and view on how to help, parents with learning disabilities, compared to for example the Norwegian system.

The results show that institutional context shed light on differences in how populations favour government interventions and is a finding that are in line with other population studies (see above under theory). Our third hypothesis is thus overall confirmed, showing that people in child’s rights protective systems favour more state interventions than those in child well-being protective systems or child maltreatment protective systems. However, findings show that the difference in responses between people under a well-being protective system and those in maltreatment systems is very small.

The findings on individual characteristic of people and their view on restricting parental rights display as expected a heterogeneity, and in line with other studies on child protective intervention there are not a consistent pattern in correlations (see Skivenes, 2023; Berrick et al., 2022). Similar findings are evident in studies about citizens’ trust in governments and public institutions (Zmerli & van der Meer, 2017), as well as in welfare state research (Svallfors, 2012). An interesting finding is the positive correlation between trust in government and the willingness to restrict parental freedom, but this is also an effect in our study that is difficult to isolate from opinions about parental behaviour. Although the results on demographic variables are not very strong, it is recognisable that respondents that suggest no state intervention are more likely to be men, having little education and being religious, but not supportive of the political orientation of the present government. Such results are also in line with findings in other studies of confidence in the child protection system (Juhasz & Skivenes, 2016; Skivenes & Benbenishty, 2023).

## Concluding remarks

In light of the ongoing European debates about child protection and how controversial and contested this area of the welfare state seem to be, it is interesting to learn (also) from this study that people, across countries, individual differences, child protection systems, overall are supportive of state intervention and support in a situation as the described vignette portrays with a child at potential risk. This indicates that the harsh critique of child protection does not include the majority of people in societies nor for not having a state intervention in families, and we may also speculate that the group of critics are quite small. Two of the suggestions for further research are related to the experiment. First, learning the impact of parental situations on the acceptance for type on intervention, provides insight into both the sentiments around personal responsibilities (substance misuse versus a condition of learning disability) should be further pursued and tested how fare this research and if there are differences in country policies about problematic parental conditions that may be treated versus non-treatable conditions. Second, it seems important to pursue if and how respondents’ views on the status of a newborn baby’s life situation up against parental struggles and problems, as it also makes sense to regard the situation as equally problematic and risky in the three scenarios that are described. Finally, we also see a need to increase the knowledge base on the role of institutional context on citizens’ views on public policies.

## Supplementary Material

Supplemental Material

## Notes

1 Policy theory has two related branches: *policy feedback theory* (policies affects politics over time, e.g. Beland, 2010) and *policy responsiveness theory* (politicians are aware and incentivised by popular preferences for policies, e.g. Brooks & Manza, 2006). Both primarily regard public opinion as an independent variable that explains, or has an impact on, politicians and in turn how policy is developed, decided, and implemented.

2 For exploratory purposes, the survey also included questions about attitudes regarding well-being and family interventions. These data are not the focus of this paper.

## Open access

With license CC BY-NC.

## References

[CIT0501] Béland, D. (2010). Reconsidering Policy Feedback: How Policies Affect Politics. *Administration & Society*, *42*(5), 568–590. 10.1177/0095399710377444

[CIT0001] Benbenishty, R., & Schmid, H. (2013). Public attitudes toward the identification and reporting of alleged maltreatment cases among social groups in Israel. *Children and Youth Services Review*, *35*(1), 332–339. 10.1016/j.childyouth.2012.11.013

[CIT0502] Berrick, J. D. (2011). Trends and issues in the U.S. child welfare system. In N. Gilbert, N. Parton, & M. Skivenes (Eds.), *Child Protection Systems: International Trends and Orientations* (pp. 17–35). New York, NY: Oxford University Press.

[CIT0504] Berrick, J., Dickens, J., Pösö, T., & Skivenes, M. (2015). Children’s involvement in care order decision-making: A cross-country analysis. *Child Abuse & Neglect*, *49*, 128–141. 26232058 10.1016/j.chiabu.2015.07.001

[CIT0503] Berrick, J., Dickens, J., Pösö, T., & Skivenes, M. (2016). Parents’ involvement in care order decisions: A cross-country study of front-line practice. *Child and Family Social Work**,* *22*(2): 626–637.

[CIT0505] Berrick, J., Dickens, J., Pösö, T. & Skivenes, M. (2019). Children’s and parents’ involvement in care order proceedings: a cross-national comparison of judicial decision-makers’ views and experiences. *Journal of Social Welfare and Family Law* *41*(2), 188-204.

[CIT0506] Berrick, J., Skivenes, M. & Roscoe, J. N. (2022). Parental freedom in the context of risk to the child: Citizens' views of child protection and the state in U.S. and Norway. *Journal of Social Policy*, 1-22. 10.1017/S0047279421001021

[CIT0002] Berrick, J. D., Gilbert, N., & Skivenes, M. (2023). *Handbook of international child protection systems*. Oxford University Press.

[CIT0507] Brooks, C., & Manza, J. (2006). Social Policy Responsiveness in Developed Democracies. *American Sociological Review*, *71*(3), 474–494. 10.1177/000312240607100306

[CIT0003] Burns, K., Helland, H., Križ, K., Segado, S., Skivenes, M., & Strömpl, J. (2021). Corporal punishment and reporting to child protection authorities: An empirical study of population attitudes in five European countries. *Children and Youth Service Review*, *120*, Article 105749. 10.1016/j.childyouth.2020.105749

[CIT0004] Convention on the Rights of the Child. (1989). Online. 27531 UNTS 1577, opened for signature 20 November 1989, entered into force 2 September 1990. https://treaties.un.org//doc/publication/UNTS/Volume%202515/v2515.pdf.

[CIT0005] Diepeveen, S., Ling, T., Suhrcke, M., Roland, M., & Marteau, T. M. (2013). Public acceptability of government intervention to change health-related behaviours: A systematic review and narrative synthesis. *BMC Public Health*, *13*(1), 756–767. 10.1186/1471-2458-13-756 23947336 PMC3765153

[CIT0006] Gilbert, N., Parton, N., & Skivenes, M. (2011). *Child protection systems: International trends and emerging orientations*. Oxford University Press.

[CIT0007] Harring, N. (2018). Trust and state intervention: Results from a Swedish survey on environmental policy support. *Environmental Science and Policy*, *82*(January), Elsevier: 1–8. 10.1016/j.envsci.2018.01.002

[CIT0008] Helland, H. S., Pedersen, S. H., & Skivenes, M. (2020). Adopsjon eller offentlig omsorg? En studie av befolkningens syn på adopsjon som tiltak i barnevernet (Adoption or public care? A study of the population’s view on adoptions from care). *Tidsskrift for Samfunnsforskning*, *61*(2), 124–139. 10.18261/issn.1504-291X-2020-02-02

[CIT0009] Hestbæk, A.-D., Skivenes, M., Falch-Eriksen, A., Svendsen, I., & Bache-Hansen, E. (2023). The child protection system in Denmark and Norway. In J. D. Berrick, N. Gilbert, & M. Skivenes (Eds.), *Oxford handbook of international child protection systems* (pp. 112–134). Oxford University Press.

[CIT0508] Höjer, I., & Pösö, T. (2023). Child protection in finland and sweden. In J. Duerr Berrick, N. Gilbert, & M. Skivenes (Eds.), *Oxford Handbook of Child Protection Systems* (pp. 156-173). Oxford University Press. 10.1093/oxfordhb/9780197503546.013.6

[CIT0509] Juhasz, I., & Skivenes, M. (2016). The population’s confidence in the child protection system – a survey study of England, Finland, Norway and the United States (California). *Social Policy and Administration*, *51*(7), 1330–1347.

[CIT0510] Kriz, K., & Skivenes, M. (2013). Systemic differences in views on risk: A comparative case vignette study of risk assessment in England, Norway and the United States (California). *Children and Youth Sciences Review*, *35*(11), 1862–1870.

[CIT0511] Kriz, K., & Skivenes, M. (2014). Street-level policy aims of child welfare workers in England, Norway and the United States: An exploratory study. *Children and Youth Service Review*, *40*, 71–78.

[CIT0512] Kriz, K., & Skivenes, M. (2015). Challenges for marginalized minority parents in different welfare systems: Child welfare workers’ perspectives. *International Social Work*, *58*(1), 75–87.

[CIT0010] Luhamaa, K., McEwan-Strand, A., Ruiken, B., Skivenes, M., & Wingens, F. (2021). Services and support to mothers and newborn babies in vulnerable situations. A study of eight countries. *Children and Youth Service Review*, *120*, Article 105762. 10.1016/j.childyouth.2020.105762

[CIT0011] Mill, J. S. (1903). *On liberty*. Watts.

[CIT0012] Parton, N., & Berridge, D. (2011). Child protection in England. In N. Gilbert, N. Parton, & M. Skivenes (Eds.), *Child protection systems: International trends and orientations* (pp. 60–87). Oxford University Press.

[CIT0013] Pösö, T. (2011). Combatting child abuse in Finland: From family to child-centered orientation. In N. Gilbert, N. Parton, & M. Skivenes (Eds.), *Child protection systems: International trends and orientations* (pp. 112–130). Oxford University Press.

[CIT0014] Regulation (EU) 2016/679. (General Data Protection Regulation), OJ L 119.

[CIT0015] Rothstein, B., & Stolle, D. (2007). The quality of government and social capital: A theory of political institutions and generalized trust. QoG Working Paper, 2.

[CIT0513] Ruiken, B. (2022). Analyzing decision-maker’s justifications of care orders for newborn children: equal and individualized treatment, *Journal of Public Child Welfare*. Advance online publication. 10.1080/15548732.2022.2158990 PMC10763567 38179110

[CIT0016] Schmid, H., & Benbenishty, R. (2011). Public attitudes toward child maltreatment in Israel: Implications for policy. *Children and Youth Services Review*, *33*(7), 1181–1188. 10.1016/j.childyouth.2011.02.015

[CIT0017] Shapiro, I. (1999). *Democratic justice*. Yale University Press.

[CIT0514] Skivenes, M. (2011). Norway – toward a child centric perspective. In N. Gilbert, N. Parton, & M. Skivenes, (Eds.), *Child protection systems: International trends and emerging orientations* (pp. 153–182). New York, NY: Oxford University Press.

[CIT0515] Skivenes, M. (2023) Exploring populations view on thresholds and reasons for child protection intervention – comparing England, Norway, Poland and Romania. *European Journal of Social Work*, *26*(1), 92-107. 10.1080/13691457.2021.1995706

[CIT0516] Skivenes, M., & Benbenishty, R. (2023). Securing permanence for children in care: A cross-country analysis of citizen's view on adoption versus foster care. *Child & Family Social Work*, *28*(2), 432– 442. 10.1111/cfs.12974

[CIT0517] Skivenes, M., & Thoburn, J. (2017). Citizens’ views in four jurisdictions on placement policies for maltreated children. *Child and Family Social Work*, *22*(4), 1472–1479.

[CIT0018] Svallfors, S. (1996). National differences in national identities? An introduction to the International Social Survey Programme. *Journal of Ethnic and Migration Studies*, *22*(1), 127–134. 10.1080/1369183X.1996.9976526

[CIT0019] Svallfors, S. (2012). *Contested welfare states: Welfare attitudes in Europe and beyond*. Stanford University Press.

[CIT0020] Thoburn, J. (2023). Child welfare and child protection services in England. In J. D. Berrick, N. Gilbert, & M. Skivenes (Eds.), *International handbook of child protection systems* (pp. 59–76). Oxford University Press.

[CIT0021] Valarino, I., Duvander, A. Z., Haas, L., & Neyer, G. (2018). Exploring leave policy preferences: A comparison of Austria, Sweden, Switzerland, and the United States. *Social Politics: International Studies in Gender, State & Society*, *25*(1), 118–147. 10.1093/sp/jxx020

[CIT0022] Young, I. (1997). *Intersecting voices: Dilemmas of gender, political philosophy, and policy*. Princeton University Press.

[CIT0023] Zmerli, S., & van der Meer, T. W. G. (2017). *Handbook on political trust*. Edward Elgar Publishing.

