# Supplementary material for: Restricting family life - an examination of citizens’ views on state interventions and parental freedom in eight European countries
Source: Eur J Soc Work. 2023 Jul 10;27(3):490–504. doi: 10.1080/13691457.2023.2227772 (PMC11057843; doi:10.1080/13691457.2023.2227772)
Supplement: Supplemental Material [file CESW_A_2227772_SM3487.docx]

**Appendix – 8 country paper. supplementary material**

**Table A1** Distribution of respondent characteristics per the randomly assigned treatment group. Total and per country (Percent and N in Parenthesis)

|  | | Gender | | Age | | | Partner | | Children | | Education | |
| --- | --- | --- | --- | --- | --- | --- | --- | --- | --- | --- | --- | --- |
|  | Treatments | Female | Male | Young | Adult | Old | Yes | No | Yes | No | Low | High |
| Austria | X1 | 33 (184) | 33 (159) | 32 (82) | 34 (130) | 33 (131) | 34 (294) | 30 (51) | 32 (204) | 36 (140) | 33 (234) | 33 (96) |
|  | X2 | 32 (178) | 34 (164) | 31 (79) | 35 (134) | 33 (130) | 34 (293) | 30 (52) | 31 (201) | 36 (142) | 33 (229) | 36 (104) |
|  | X3 | 34 (190) | 33 (162) | 37 (94) | 31 (119) | 34 (138) | 32 (282) | 40 (67) | 37 (241) | 28 (110) | 34 (238) | 31 (92) |
| England | X1 | 30 (279) | 36 (295) | **38 (186)** | 33 (207) | **29 (182)** | 31 (346) | 37 (227) | 33 (416) | 33 (157) | 33 (263) | 33 (293) |
|  | X2 | 35 (325) | 31 (253) | **26 (125)** | 32 (201) | **40 (249)** | 36 (402) | 28 (174) | 34 (428) | 31 (145) | 34 (269) | 32 (284) |
|  | X3 | 34 (316) | 33 (276) | **36 (172)** | 35 (223) | **31 (196)** | 34 (377) | 35 (212) | 33 (419) | 36 (169) | 32 (255) | 34 (300) |
| Estonia | X1 | 29 (163) | 36 (148) | 34 (84) | 31 (109) | 31 (117) | 32 (136) | 32 (172) | 34 (25) | 32 (292) | 32 (137) | 32 (149) |
|  | X2 | 38 (217) | 29 (116) | 36 (89) | 33 (115) | 33 (126) | 37 (158) | 32 (169) | 29 (22) | 35 (316) | 35 (152) | 34 (158) |
|  | X3 | 33 (188) | 35 (142) | 30 (72) | 36 (124) | 35 (133) | 31 (130) | 36 (194) | 37 (27) | 34 (308) | 33 (143) | 34 (161) |
| Finland | X1 | 34 (172) | 33 (166) | 37 (88) | 33 (118) | 32 (131) | 39 (151) | 30 (184) | 33 (249) | 33 (88) | 33 (215) | 35 (120) |
|  | X2 | 35 (181) | 32 (159) | 32 (78) | 33 (120) | 35 (141) | 31 (118) | 35 (217) | 33 (243) | 37 (96) | 34 (223) | 33 (114) |
|  | X3 | 31 (157) | 35 (176) | 31 (75) | 34 (123) | 33 (136) | 30 (117) | 35 (212) | 34 (254) | 30 (78) | 33 (217) | 32 (112) |
| Germany | X1 | 33 (356) | 34 (329) | 33 (159) | 35 (261) | 32 (266) | 34 (571) | 33 (107) | 33 (447) | 34 (237) | 34 (460) | 34 (188) |
|  | X2 | 34 (371) | 32 (306) | 31 (150) | 34 (254) | 33 (272) | 34 (572) | 27 (89) | 33 (444) | 33 (230) | 32 (435) | 33 (184) |
|  | X3 | 33 (365) | 34 (330) | 36 (173) | 32 (237) | 34 (283) | 32 (548) | 40 (133) | 34 (461) | 33 (233) | 35 (474) | 33 (181) |
| Ireland | X1 | 33 (176) | 33 (161) | **28 (80)** | 36 (154) | 33 (106) | 32 (228) | 33 (108) | 31 (173) | 34 (165) | 34 (140) | 33 (179) |
|  | X2 | 34 (183) | 34 (169) | **36 (102)** | 34 (149) | 33 (104) | 34 (239) | 35 (114) | 35 (192) | 33 (162) | 33 (139) | 33 (179) |
|  | X3 | 33 (177) | 33 (164) | **36 (103)** | 30 (130) | 34 (108) | 34 (238) | 32 (104) | 34 (186) | 32 (156) | 33 (137) | 34 (182) |
| **Norway** | **X1** | **31 (235)** | **37 (267)** | **32 (134)** | **33 (185)** | **36 (185)** | **34 (500)** | **34 (9)** | **33 (317)** | **35 (186)** | **34 (296)** | **34 (208)** |
|  | X2 | 34 (258) | 32 (230) | 31 (130) | 34 (187) | 33 (172) | 33 (487) | **24 (6)** | 33 (317) | 32 (170) | 33 (295) | 32 (193) |
|  | X3 | 36 (272) | 31 (228) | 37 (158) | 33 (182) | 31 (160) | 33 (494) | **41 (11)** | 34 (326) | 33 (172) | 33 (292) | 34 (208) |
| Spain | X1 | 35 (187) | 32 (150) | 34 (73) | 35 (147) | 31 (117) | 35 (284) | 29 (53) | 33 (198) | 35 (133) | 31 (137) | 32 (78) |
|  | X2 | 34 (182) | 33 (153) | 37 (80) | 32 (134) | 33 (122) | 33 (269) | 36 (67) | 33 (202) | 32 (125) | 34 (149) | 33 (80) |
|  | X3 | 31 (166) | 36 (168) | 30 (65) | 32 (135) | 36 (136) | 33 (269) | 36 (66) | 34 (205) | 33 (127) | 35 (154) | 35 (85) |
| Total | X1 | 32 (1752) | 34 (1674) | 34 (886) | 34 (1311) | 32 (1235) | 33 (2510) | 33 (912) | 33 (2029) | 34 (1398) | 33 (1883) | 33 (1312) |
|  | X2 | 35 (1896) | 32 (1550) | 32 (831) | 33 (1294) | 34 (1317) | 34 (2538) | 32 (888) | 33 (2049) | 34 (1386) | 33 (1890) | 33 (1296) |
|  | X3 | 33 (1830) | 34 (1645) | 35 (912) | 33 (1272) | 34 (1289) | 33 (2455) | 36 (1000) | 34 (2118) | 33 (1354) | 34 (1910) | 34 (1319) |

**Table A1** Distribution of respondent characteristics per the randomly assigned treatment group. Total and per country (Percent and N in Parenthesis)

|  |  | Religiouness | | Income | | | Immigrant | | Political Affiliation | |
| --- | --- | --- | --- | --- | --- | --- | --- | --- | --- | --- |
|  | Treatments | Religious | Non-Religous | Low | Middle | High | No | Yes | Government | Oppostion |
| Austria | X1 | 37 (233) | **27 (93)** | 30 (51) | 32(125) | 35 (168) | 35 (324) | 19 (20) | 34 (137) | 34 (117) |
|  | X2 | 31 (197) | **37 (128)** | 36 (61) | 31(121) | 33 (161) | 31 (293) | 47 (48) | 33 (132) | 32 (110) |
|  | X3 | 32 (207) | **36 (124)** | 33 (56) | 37(143) | 32 (153) | 34 (314) | 34 (35) | 33 (131) | 34 (118) |
| England | X1 | 32 (244) | 35 (312) | 35 (80) | 33(140) | 33 (356) | 34 (534) | 30 (28) | 33 (159) | 34 (287) |
|  | X2 | 36 (277) | 31 (276) | 33 (76) | 31(132) | 34 (369) | 33 (530) | 34 (32) | 35 (170) | 31 (264) |
|  | X3 | 32 (245) | 35 (310) | 31 (71) | 36 (151) | 34 (369) | 33 (519) | 36 (34) | 31 (152) | 35 (293) |
| Estonia | X1 | **29 (71)** | 34 (218) | 32 (193) | 34 (73) | 29 (44) | 32 (307) | **51 (3)** | **30 (91)** | **35 (191)** |
|  | X2 | **31 (75)** | 34 (223) | 34 (204) | 31 (66) | 39 (59) | 34 (327) | **41 (3)** | **39 (119)** | **29 (161)** |
|  | X3 | **39 (95)** | 32 (208) | 34 (205) | 34 (73) | 32 (50) | 34 (325) | **8 (1)** | **31 (96)** | **36 (193)** |
| Finland | X1 | 33 (171) | 35 (151) | 31 (135) | 37 (116) | 32 (88) | 33 (327) | **42 (8)** | 32 (111) | 34 (122) |
|  | X2 | 34 (175) | 33 (140) | 35 (148) | 33 (102) | 33 (89) | 34 (330) | **26 (5)** | 34 (117) | 33 (118) |
|  | X3 | 34 (176) | 32 (136) | 34 (146) | 30 (94) | 35 (94) | 33 (321) | **32 (6)** | 34 (115) | 34 (121) |
| Germany | X1 | 34 (369) | 33 (273) | 30 (129) | 34 (210) | 34 (347) | 33 (605) | 34 (54) | 35 (239) | 33 (275) |
|  | X2 | 33 (366) | 32 (261) | 31 (130) | 32 (193) | 34 (353) | 32 (594) | 33 (52) | 31 (214) | 33 (276) |
|  | X3 | 33 (362) | 35 (293) | 39 (164) | 34 (209) | 32 (323) | 34 (631) | 33 (51) | 34 (231) | 34 (278) |
| Ireland | X1 | 32 (228) | **34 (92)** | 30 (36) | 39 (80) | 31 (222) | 31 (238) | **38 (90)** | 34 (110) | 32 (117) |
|  | X2 | 32 (226) | **40 (109)** | 34 (41) | 29 (60) | 36 (252) | 34 (263) | **34 (81)** | 33 (107) | 36 (131) |
|  | X3 | 36 (252) | **26 (72)** | 36 (43) | 32 (65) | 33 (233) | 34 (262) | **28 (66)** | 33 (106) | 32 (115) |
| Norway | X1 | 35 (244) | 32 (234) | 32 (275) | 36 (138) | 36 (90) | 34 (479) | 34 (25) | 38 (233) | 30 (257) |
|  | X2 | 35 (245) | 30 (217) | 33 (284) | 31 (119) | 34 (84) | 33 (466) | 32 (23) | 30 (181) | 35 (298) |
|  | X3 | 30 (214) | 38 (272) | 34 (294) | 34 (132) | 30 (73) | 33 (476) | 33 (24) | 33 (199) | 34 (289) |
| Spain | X1 | 32 (180) | 35 (129) | 42 (30) | 32 (104) | 33 (202) | 33 (313) | 35 (22) | 35 (84) | 33 (250) |
|  | X2 | 34 (186) | 32 (119) | 33 (24) | 38 (124) | 31 (188) | 33 (312) | 29 (18) | 32 (78) | 34 (254) |
|  | X3 | 34 (187) | 34 (125) | 25 (18) | 30 (100) | 36 (217) | 33 (311) | 37 (24) | 33 (80) | 33 (252) |
| Total | X1 | 33 (1739) | 33 (1500) | 32 (928) | 34(986) | 33 (1517) | 33 (3128) | 33 (250) | 34 (1163) | 33 (1617) |
|  | X2 | 33 (1748) | 33 (1472) | 33 (969) | 32(918) | 34 (1553) | 33 (3113) | 35 (263) | 33 (1117) | 33 (1610) |
|  | X3 | 33 (1738) | 34 (1539) | 34 (997) | 34(966) | 33 (1512) | 34 (3158) | 32 (241) | 33 (1110) | 34 (1659) |

**Table A2** Responses to Vignette total and per country. Percent and N in parenthesis. Total N=10,348

|  | (1) | (2) | (3) | (4) | (5) | Total |
| --- | --- | --- | --- | --- | --- | --- |
| Austria | 3.49 (36) | 42.46 (441) | 45.24 (470) | 4.66 (48) | 4.15 (43) | 100 (1038) |
| England | 2.66 (46) | 34.71 (605) | 43.91 (765) | 12.33 (215) | 6.40 (111) | 100 (1743) |
| Estonia | 2.57 (25) | 41.43 (402) | 45.50 (441) | 5.98 (58) | 4.51 (44) | 100 (970) |
| Finland | 4.18 (42) | 31.01 (314) | 47.12 (477) | 12.14 (123) | 5.55 (56) | 100 (1013) |
| Germany | 6.07 (125) | 33.25 (684) | 47.50 (977) | 7.92 (163) | 5.26 (108) | 100 (2057) |
| Ireland | 4.27 (44) | 37.25 (384) | 45.75 (472) | 10.02 (103) | 2.71 (28) | 100 (1032) |
| Norway | 1.34 (20) | 25.68 (383) | 50.70 (756) | 13.03 (194) | 9.25 (138) | 100 (1492) |
| Spain | 4.08 (41) | 35.36 (355) | 47.59 (478) | 8.34 (84) | 4.63 (46) | 100 (1005) |
| Total | 3.67 (380) | 34.48 (3568) | 46.74 (4837) | 9.55 (989) | 5.56 (575) | 100 (10348) |

### **Tabell A3a: Significant differences between countries on “No restrictions” (value 1). Zigne. Simple random sample, one-sided test (1%)**

|  | Ireland | Austria | UK | Estonia | Finland | Spain | Germany | Norway |
| --- | --- | --- | --- | --- | --- | --- | --- | --- |
| Ireland |  |  | X |  |  |  |  | X |
| Austria |  |  |  |  |  |  | X | X |
| UK | X |  |  |  | X | X | X |  |
| Estonia |  |  |  |  |  |  | X |  |
| Finland |  |  | X |  |  |  |  | X |
| Spain |  |  | X |  |  |  |  | X |
| Germany |  | X | X | X |  |  |  | X |
| Norway | X | X |  |  | X | X | X |  |

### **Tabell A3b: Significant differences between countries. Some restrictions (merged value 2 and 3). Zigne. Simple random sample, one-sided test (1%)**

|  | Ireland | Austria | UK | Estonia | Finland | Spain | Germany | Norway |
| --- | --- | --- | --- | --- | --- | --- | --- | --- |
| Ireland |  | X | X | X | X |  |  | X |
| Austria | X |  | X |  | X | X | X | X |
| UK | X | X |  | X |  | X |  |  |
| Estonia | X |  | X |  | X | X | X | X |
| Finland | X | X |  | X |  | X |  |  |
| Spain |  | X | X | X | X |  |  | X |
| Germany |  | X |  | X |  |  |  | X |
| Norway | X | X |  | X |  | X | X |  |

### **Tabell A3c: Significant differences between countries. Removal of child (merged value 4 and 5). Zigne. Simple random sample, one-sided test (1%)**

|  | Ireland | Austria | UK | Estonia | Finland | Spain | Germany | Norway |
| --- | --- | --- | --- | --- | --- | --- | --- | --- |
| Ireland |  | X | X |  | X |  |  | X |
| Austria | X |  | X |  | X | X | X | X |
| UK | X | X |  | X |  | X | X |  |
| Estonia |  |  | X |  | X |  | X | X |
| Finland | X | X |  | X |  | X | X | X |
| Spain |  | X | X |  | X |  |  | X |
| Germany |  | X | X | X | X |  |  | X |
| Norway | X | X |  | X | X | X | X |  |

**Table A4** Mean values and standard deviation (SD), on responses to treatments X1-X3. Per country and total sample.

|  | Treatments | Mean | SD |
| --- | --- | --- | --- |
| Austria | X1: Substance X1 | 2.73 | 0.84 |
|  | X2: Mental X2 | 2.64 | 0.84 |
|  | X3: Learning X3 | 2.53 | 0.71 |
| England | X1: Substance X1 | 3.04 | 0.90 |
|  | X2: Mental X2 | 2.89 | 0.88 |
|  | X3: Learning X3 | 2.63 | 0.88 |
| Estonia | X1: Substance X1 | 2.94 | 0.90 |
|  | X2: Mental X2 | 2.71 | 0.80 |
|  | X3: Learning X3 | 2.42 | 0.64 |
| Finland | X1: Substance X1 | 3.06 | 0.91 |
|  | X2: Mental X2 | 2.78 | 0.88 |
|  | X3: Learning X3 | 2.67 | 0.84 |
| Germany | X1: Substance X1 | 2.90 | 0.93 |
|  | X2: Mental X2 | 2.69 | 0.86 |
|  | X3: Learning X3 | 2.60 | 0.85 |
| Ireland | X1: Substance X1 | 2.84 | 0.80 |
|  | X2: Mental X2 | 2.66 | 0.80 |
|  | X3: Learning X3 | 2.59 | 0.82 |
| Norway | X1: Substance X1 | 3.15 | 0.91 |
|  | X2: Mental X2 | 2.98 | 0.84 |
|  | X3: Learning X3 | 2.96 | 0.93 |
| Spain | X1: Substance X1 | 2.85 | 0.90 |
|  | X2: Mental X2 | 2.81 | 0.84 |
|  | X3: Learning X3 | 2.56 | 0.77 |
| Total | X1: Substance X1 | 2.95 | 0.90 |
|  | X2: Mental X2 | 2.78 | 0.85 |
|  | X3: Learning X3 | 2.63 | 0.84 |

**Table A4a** Mean values and SD, whole sample. Total n= 10,348.

|  | N | Mean | Standard Deviation |
| --- | --- | --- | --- |
| Austria | 1033 | 2.64 | 0.80 |
| England | 1735 | 2.85 | 0.90 |
| Estonia | 1012 | 2.68 | 0.81 |
| Finland | 1008 | 2.84 | 0.89 |
| Germany | 2047 | 2.73 | 0.89 |
| Ireland | 1027 | 2.70 | 0.81 |
| Norway | 1486 | 3.03 | 0.90 |
| Spain | 1000 | 2.74 | 0.85 |
| Total | 10348 | 2.79 | 0.87 |

**Table** A5: Comparisons of Mean Willingness by Treatments Using Tukey Post Hoc Test

|  |  |  |  |  |  |  | 99% Confidence Interval | |
| --- | --- | --- | --- | --- | --- | --- | --- | --- |
|  | Treatments | Mean | *F*-Test | Mean Differences | | Significance | Lower Bond | Upper Bond |
| Austria | X1: Substance abuse | 2.73 | *F*(2,1030)=5.55** | X2 vs X1 | –0.07 | 0.452 | –0.26 | 0.11 |
|  | X2: Mental health | 2.64 |  | X3 vs X1*** | –0.20 | 0.000 | –0.44 | –0.07 |
|  | X3: Learning disability | 2.53 |  | X3 vs X2 | –0.13 | 0.434 | –0.26 | 0.10 |
| England | X1: Substance abuse | 3.04 | *F*(2,1732)=30.96*** | X2 vs X1* | –0.15 | 0.011 | –0.30 | 0.11 |
|  | X2: Mental health | 2.89 |  | X3 vs X1*** | –0.39 | 0.000 | –0.54 | –0.24 |
|  | X3: Learning disability | 2.63 |  | X3 vs X2*** | –0.24 | 0.000 | –0.39 | 0.09 |
| Estonia | X1: Substance abuse | 2.94 | *F*(2,1009)=37.18*** | X2 vs X1** | –0.21 | 0.001 | –0.39 | –0.04 |
|  | X2: Mental health | 2.71 |  | X3 vs X1*** | –0.45 | 0.000 | –0.62 | –0.27 |
|  | X3: Learning disability | 2.41 |  | X3 vs X2*** | –0.23 | 0.000 | –0.41 | –0.06 |
| Finland | X1: Substance abuse | 3.06 | *F*(2,1005)=17.94*** | X2 vs X1* | –0.28 | 0.011 | –0.48 | 0.08 |
|  | X2: Mental health | 2.78 |  | X3 vs X1*** | –0.40 | 0.000 | –0.60 | –0.20 |
|  | X3: Learning disability | 2.67 |  | X3 vs X2 | –0.12 | 0.198 | –0.31 | –0.08 |
| Germany | X1: Substance abuse | 2.90 | *F*(2, 2044)= 20.89*** | X2 vs X1*** | –0.22 | 0.000 | –0.36 | –0.08 |
|  | X2: Mental health | 2.69 |  | X3 vs X1*** | –0.32 | 0.000 | –0.45 | –0.18 |
|  | X3: Learning disability | 2.60 |  | X3 vs X2 | –0.09 | 0.116 | –0.23 | 0.04 |
| Ireland | X1: Substance abuse | 2.84 | *F*(2,1024)=9.15*** | X2 vs X1* | –0.18 | 0.011 | –0.36 | –0.08 |
|  | X2: Mental health | 2.66 |  | X3 vs X1*** | –0.25 | 0.000 | –0.46 | –0.18 |
|  | X3: Learning disability | 2.58 |  | X3 vs X2 | –0.08 | 0.434 | –0.23 | 0.04 |
| Norway | X1: Substance abuse | 3.15 | *F*(2,1483)=6.85** | X2 vs X1** | –0.17 | 0.009 | –0.33 | 0.00 |
|  | X2: Mental health | 2.98 |  | X3 vs X1** | –0.18 | 0.003 | –0.35 | –0.02 |
|  | X3: Learning disability | 2.96 |  | X3 vs X2 | –0.02 | 0.948 | –0.18 | 0.15 |
| Spain | X1: Substance abuse | 2.85 | *F*(2,997)=11.38*** | X2 vs X1 | –0.05 | 0.700 | –0.24 | 0.14 |
|  | X2: Mental health | 2.81 |  | X3 vs X1*** | –0.29 | 0.000 | –0.48 | –0.10 |
|  | X3: Learning disability | 2.56 |  | X3 vs X2** | –0.24 | 0.001 | –0.43 | –0.05 |
| Total | X1: Substance abuse | 2.95 | *F*(2,10345)=117.02*** | X2 vs X1*** | –0.17 | 0.000 | –0.36 | 0.00 |
|  | X2: Mental health | 2.78 |  | X3 vs X1*** | –0.31 | 0.000 | –0.44 | –0.07 |
|  | X3: Learning disability | 2.63 |  | X3 vs X2*** | –0.14 | 0.000 | –0.26 | 0.10 |
| **Note:** **p*≤.05; ***p*≤.01; and ****p*≤.001. | | | | | | | | |

**Table A6** Distribution of respondent characteristics per response category. Per total sample. Percent and n in parenthesis. In italics differences of 20% within category assumes to be evenly distributed.

|  | Response values | Gender | | Age | | | Partner | | Children | | Education | |
| --- | --- | --- | --- | --- | --- | --- | --- | --- | --- | --- | --- | --- |
|  |  | Female | Male | Young | Adult | Old | Yes | No | No | Yes | Low | High |
| **Total** | **1** | ***37 (139)*** | ***63 (241)*** | **36 (135)** | **40 (150)** | **25 (95)** | ***72 (270)*** | ***28 (103)*** | **50 (189)** | **50 (190)** | ***69 (228)*** | ***31 (105)*** |
|  | 2 | 51 (1810) | 49 (1758) | 28 (996) | 35 (1233) | 38 (1339) | 70 (2507) | 30 (1050) | 60 (2156) | 40 (1409) | 57 (1913) | 43 (1420) |
|  | 3 | 54 (2620) | 46 (2217) | 24 (1180) | 35 (1682) | 41 (1974) | 73 (3519) | 27 (1298) | 62 (2987) | 38 (1843) | 61 (2738) | 39 (1754) |
|  | 4 | 49 (488) | 51 (501) | 25 (250) | 38 (371) | 37 (367) | 71 (704) | 29 (282) | 62 (612) | 38 (375) | 57 (519) | 43 (399) |
|  | **5** | **43 (247)** | **57 (328)** | **27 (155)** | **39 (222)** | **34 (198)** | **75 (427)** | **25 (142)** | **61 (351)** | **39 (222)** | **55 (293)** | **45 (241)** |

|  | Response values | Religion | | Income | | | Political Affiliation | |
| --- | --- | --- | --- | --- | --- | --- | --- | --- |
|  |  | Religious | Non-Religous | Low | Middle | High | Government | Oppostion |
| **Total** | **1** | ***61 (209)*** | ***39 (132)*** | **30 (115)** | **29 (112)** | **40 (153)** | ***39 (116)*** | ***61 (181)*** |
|  | 2 | 54 (1799) | 46 (1557) | 27 (972) | 28 (984) | 45 (1612) | 39 (1121) | 61 (1761) |
|  | 3 | 54 (2467) | 46 (2096) | 29 (1392) | 27 (1308) | 44 (2136) | 41 (1595) | 59 (2262) |
|  | 4 | 54 (509) | 46 (432) | 28 (279) | 26 (262) | 45 (448) | 46 (354) | 54 (423) |
|  | **5** | **46 (246)** | **54 (288)** | **29 (166)** | **29 (164)** | **43 (244)** | **50 (230)** | **50 (231)** |

Table 7 display results from the regression analysis with six models. The analysis confirms that substance abuse exerts a much greater influence (b=0.32, p<0.001) on support for restricting parental freedom than mental health condition (b=0.14, p<0.001). When the country and type of child protection system are introduced as independent variables into the regression equations of Models 2 and 3, respectively, these effects stay nearly unchanged. Additionally, estimates reported under Model 3 show that citizens in the child right system are more supportive of restricting parental freedom (*b*=0.25, *p*<0.001) than those in the child maltreatment system (*b*=0.06, *p*<0.01). Together, these evidence provide extremely strong support for H3 that respondents exposed to information about parental substance abuse would favor governmental intervention to restrict parental freedom.

These evidence also support H4, suggesting that citizens in the child rights system are more likely to support reducing parental freedom than people in the child maltreatment or wellbeing systems.

Comparing the estimates reported under Models 4 through 6 suggests that while substance abuse causes people to consider limiting parental freedom across all three types of child protection systems, mental health concerns are only important in maltreatment and wellbeing systems. However, there is great variance in the strength of the impacts. Thus, substance abuse is the most prominent predictor of restricting parental freedom in the maltreatment system (b=0.41, p<0.001), followed by the child welfare system (b=0.29, p<0.001) and the child rights system (b=0.26, p<0.001). Simultaneously, mental health issue is a more prominent predictor of restricting (b=0.22, p<0.001) than in the wellbeing system (b=0.15, p<0.001) and is not a cause for concern among those in the child rights system.

| **Table 7** OLS Estimates of treatments on support for restricting freedom | | | | | | |
| --- | --- | --- | --- | --- | --- | --- |
|  | Model 1 | Model 2 | Model 3 | Model 4 Maltreatment | Model 5 Wellbeing | Model 6 Rights |
| Constant | 2.63*** | 2.88*** | 2.55*** | 2.60*** | 2.51*** | 2.85*** |
| Conditions (ref. X3: Learning disability) |  |  |  |  |  |  |
| X1: Substance abuse | 0.32*** | 0.32*** | 0.32*** | 0.41*** | 0.29*** | 0.26*** |
| X2: Mental health | 0.14*** | 0.15*** | 0.15*** | 0.22*** | 0.15*** | 0.07 |
| Gender (ref. Male) 1 |  |  |  |  |  |  |
| Male |  |  | –0.02 | –0.00 | –0.01 | –0.07† |
| Age (ref. Young) 2 |  |  |  |  |  |  |
| Adult |  |  | 0.06** | 0.06 | 0.11* | –0.00 |
| Old |  |  | 0.05* | 0.05 | 0.07 | 0.06 |
| Partner (ref. Yes) 3 |  |  |  |  |  |  |
| No |  |  | –0.07** | –0.04 | 0.03 | –0.19*** |
| Children (ref. No) 4 |  |  |  |  |  |  |
| Yes |  |  | –0.05* | –0.11** | –0.05 | 0.05 |
| Education (ref. Low) 5 |  |  |  |  |  |  |
| High |  |  | 0.04* | –0.01 | –0.04 | 0.18*** |
| Religious (ref. Yes) 6 |  |  |  |  |  |  |
| No |  |  | 0.06* | –0.00 | 0.09** | 0.06 |
| Income (ref. Low) 7 |  |  |  |  |  |  |
| Middle |  |  | –0.04 | 0.06 | 0.00 | 0.00 |
| High |  |  | 0.04 | 0.08 | 0.12** | –0.08 |
| Immigrant (ref. No) 8 |  |  |  |  |  |  |
| Yes |  |  | –0.10* | –0.15* | 0.02 | –0.23* |
| Affiliation (ref. Government Party) 9 |  |  |  |  |  |  |
| Opposition Party |  |  | –0.08*** | –0.04 | –0.13*** | –0.07* |
| Countries (ref. Norway) |  |  |  |  |  |  |
| Austria |  | –0.39*** |  |  |  |  |
| England |  | –0.18*** |  |  |  |  |
| Estonia |  | –0.34*** |  |  |  |  |
| Finland |  | –0.19*** |  |  |  |  |
| Germany |  | –0.30*** |  |  |  |  |
| Ireland |  | –0.33*** |  |  |  |  |
| Spain |  | –0.29*** |  |  |  |  |
| Child Protection System (ref. Welbeing) |  |  |  |  |  |  |
| Maltreatment |  |  | 0.06** |  |  |  |
| Rights |  |  | 0.25*** |  |  |  |
| *Adjusted R^2^* | 0.02 | 0.04 | 0.04 | 0.04 | 0.03 | 0.04 |
| N | 10,348 | 10,348 | 7,271 | 2,562 | 2,642 | 2,067 |
| **Note:** †*p*≤.10; **p*≤.05; ***p*≤.01; and ****p*≤.001. | | | | | | |
